# Study on the movement mechanism of rice stem under the action of canopy-opening device based on explicit dynamics simulation

**DOI:** 10.3389/fpls.2023.1252247

**Published:** 2023-10-24

**Authors:** Lin-long Jing, Xin-hua Wei, Qi Song, Fei Wang

**Affiliations:** Key Laboratory of Modern Agricultural Equipment and Technology, Ministry of Education of the People’s Republic of China, Institute of Agricultural Engineering, Jiangsu University, Zhenjiang, China

**Keywords:** canopy-opening device, explicit dynamics, rice, motion mechanism, canopy deposition

## Abstract

The dense canopy of rice causes attenuation of droplet dispersion during pesticide application. The canopy-opening device can increase droplet deposition in the middle and lower canopy of rice by causing disturbance to the rice canopy. However, the conditions for use of the canopy-opening device are difficult to determine. Rice morphological structure parameters and material parameters were measured to study the movement mechanism of the rice stems under the action of the canopy-opening device, and the canopy-opening process was then simulated using the explicit dynamic method. The simulation scene of the rice canopy-opening process considered the combination of three different heights and three different driving velocities of the canopy-opening device. The movement mechanism of the rice stems under the operation of the canopy-opening device was investigated, and the entire movement process was separated into two stages: contact and oscillation. The simulation results and high-speed photography experimental results show a strong correlation, with a correlation coefficient of 0.733. The simulation results indicate that when the canopy-opening device is closer to the ground and the driving velocity is higher, the disturbance to the rice stem during the contact stage is stronger. However, for the oscillation stage, there exists a critical value for both the height and driving velocity of the canopy-opening device. During the oscillation stage, there is a critical value for both the height and driving velocity of the canopy-opening device. The numerical-based explicit dynamics approach was employed in this work to investigate the rice canopy motion mechanism, and this study has a definite reference value for the investigation of complicated motion mechanisms in the field crop production process.

## 1 Introduction

When spraying pesticides, the rice canopy structure has a great influence on the spread and retention of droplets (Farooq and Salyani, 2004; Da Silva et al., 2006; Gaskin et al., 2013). A dense canopy is formed in the middle and late stages of rice growth. The permeability of spraying in the rice canopy decreases as leaf density increases (Liu et al., 2021), as does spray uniformity (Xu et al., 2021). The effect of disease prevention and treatment on the middle and lower canopy of rice will be reduced. The main diseases and insect pests affecting rice growth during the middle and late stages are sheath blight, rice planthopper, and rice blast. These pests typically affect the middle and lower canopy of the rice plant and can cause significant damage to rice production throughout the year (Lee, 1983; Savary et al., 2000; Skamnioti and Gurr, 2009; Heong et al., 2016; Liu et al., 2016). To improve the effectiveness of rice plant protection operations, it is critical to optimize the spraying method. To improve droplet penetration, and raise droplet deposition quantity in the middle and lower canopy of rice.

The canopy-opening device is a device that can cause disturbance to the crop canopy. Usually composed of conduits or pipes installed in front of the spray arm, it has the characteristics of simple structure, high reliability, and minimal mechanical damage to plants. The canopy-opening device can effectively open the internal space of the crop canopy (Wu and Wei, 2019) and is not affected by the characteristics of the canopy (Prado et al., 2016), making it easier for droplets to reach the middle and lower layers of the canopy, improving the permeability and uniformity of droplets in the crop canopy (Wang et al., 2021).

The spatial position relationship between the canopy-opening device and the spray boom is shown in **Supplementary Figure 1**. The canopy-opening device (**Supplementary Figure 1A**) is typically mounted on the lower front of the spray boom. The hydraulic system controls the lifting and lowering of the canopy-opening device and the spray boom. The distance between the canopy-opening device and the spray boom can be adjusted in the vertical direction, ranging from 0 to 0.5 meters (**Supplementary Figure 1B**), and in the horizontal direction, ranging from 0 to 0.5 meters (**Supplementary Figure 1C**). Before spraying, the canopy-opening device is adjusted to be inside the rice canopy. As the sprayer advances, the canopy-opening device pushes the movement of the rice canopy, effectively opening up the internal space of the crop canopy (Wu and Wei, 2019), making it easier for droplets to reach the middle and lower layers of the canopy and solving the problem of branch and leaf shading.

The function of the canopy-opening device can greatly enhance droplet penetration and uniformity in the crop canopy (Wang et al., 2021). It can improve droplet deposition rate by 8.6~18.0% for varied nozzles (Womac et al., 2022). However, determining the position of the canopy-opening device is challenging. If the location is too high, it is difficult to open the rice canopy; if it is too low, the rice will be harmed. There are several elements that influence canopy-opening effect, however the influence rule of each component on canopy-opening effect is unclear.

Zhu et al. established a mathematical model according to the physical characteristics of soybean plants to determine the best position of the canopy-opening device (Zhu et al., 2008). It should be noted that in this study, only greenhouse soybean was utilized as the research object, and only the location of the canopy-opening device was investigated. According to the characteristics of rice plants, Wu and Wei analyzed the canopy-opening process through transient dynamic simulation and investigated the mathematical relationship between time and displacement at different canopy-opening device positions (Wu and Wei, 2019). The influence of driving velocity on the canopy’s motion mechanism, on the other hand, had not been studied. Most of these studies had been conducted under considerations of static or quasi-static loading cases, small strain deformation, short time, small displacement, and linear contact assumptions through implicit solvers (Celik, 2017).

In solving dynamic problems, explicit finite element method (FEM) and implicit FEM are commonly used. The explicit method is essentially an incremental method used to determine the dynamic response of the structure (Celik, 2017). One advantage of the explicit process over the implicit process is that it is easier to solve complex contact problems, without iteration and convergence issues, and as the model size increases, the explicit method is more cost-effective than the implicit method. Rice canopy-opening process is a long-time (>1s), low-velocity and large displacement dynamic and complex contact process. In this context, the explicit solution approach has been pointed out to be valuable in solving loading cases such as dynamic contact events (Wakabayashi et al., 2008; SolidWorks Doc., 2010; Lee, 2012; Wu and Gu, 2012; ANSYS Doc., 2016). As a result, displacement caused by the rice canopy-opening process can be considered a nonlinear dynamics application covered by the aforementioned explicit dynamics system, and the use of explicit dynamic simulation can well reveal the kinematic characteristics of stamping, collision, drop, etc.

The contribution of this study is to simulate the process of rice canopy-opening through the explicit dynamic simulation method, and to study the movement mechanism of the rice canopy under the action of the canopy-opening device. Rice morphological structure parameters and material parameters were measured, and a relationship model was established between the position and driving velocity of the canopy-opening device and the maximum displacement of the canopy-opening process in this study. The relationship model was tested by high-speed photography. It may be used as a reference point for simulations of different crops as well as the optimization of canopy-opening device, installation location, sinking position, and driving velocity. Provided an optimization idea for improving the droplet permeability in dense crop canopy.

## 2 Materials and methods

Select 300 disease- and insect-free rice plants at the heading stage on the RunGuo Agricultural Base in Zhenjiang City in Jiangsu Province (32°54′19′′N, 116°23′28′′E) on Sep 6, 2021. The rice varieties studied were ‘Longliangyou 2010’ (**Supplementary Figure 2**). The row spacing of rice plants was 18 cm and the plant spacing per row was 10 cm. The temperature was 26.27°C, the humidity was 75.38%. The collected rice was used for the measurement of rice morphological structure parameters and material parameters, and the relevant detection measurements were completed within 24 hours.

### 2.1 The simulation model of rice canopy-opening process

Rice morphological structure parameters and material parameters were used as the basis for explicit dynamics simulation. All measurements and tests of morphological structure parameters and material parameters were carried out at the physical property analysis laboratory of the Agricultural Engineering Institute (Jiangsu University, Zhenjiang, China). The morphological structure parameters of the canopy-opening device and rice were taken for modelling the canopy-opening device and rice geometry as the first step. A simulation model of the rice canopy process was created using the material parameters and geometric models of the rice plant and the canopy-opening device. The simulation model of the rice canopy-opening process was simulated after modeling. The simulation results were post-processed, and the movement mechanism of the rice canopy was analyzed.

#### 2.1.1 Measurement of morphological structure parameters

**Figure 1:**
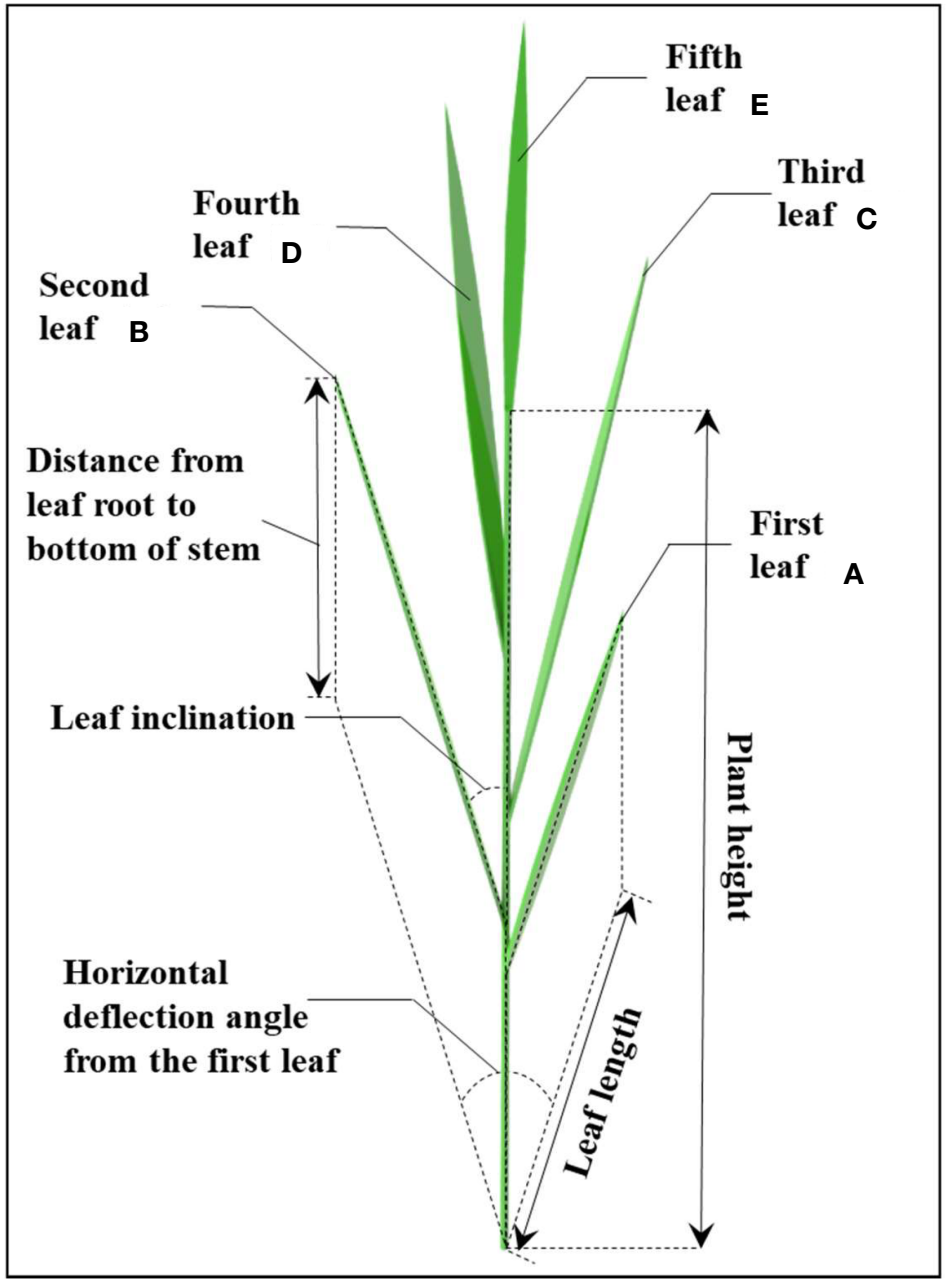
The geometric relation and spatial distribution of rice stem and leaves.

To establish a geometry structure model of rice and canopy-opening device, the morphological structure parameters of rice plants and canopy-opening device must be determined experimentally. The parameters (plant height, number of leaves, leaf area, leaf width, stem length, stem diameter, stem wall thickness) of rice plants were measured (**Figure 1**) (Shu and Wei, 2019). To simplify the rice structure, the stem and leaves are considered primarily. Rice morphological structural parameters were measured and averaged using 100 selected rice plants.

**Figure 2:**
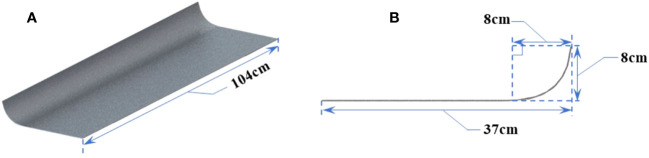
The geometric model and morphological parameters of canopy-opening device. **(A)** Oblique view, **(B)** Side view.

Some measurements of morphological structure parameters on the whole rice plant and the test results are shown in **Supplementary Table 1**. The geometric model and morphological parameters of canopy-opening device are shown in the **Figure 2**.

#### 2.1.2 Measurement of rice material parameters

**Figure 3:**
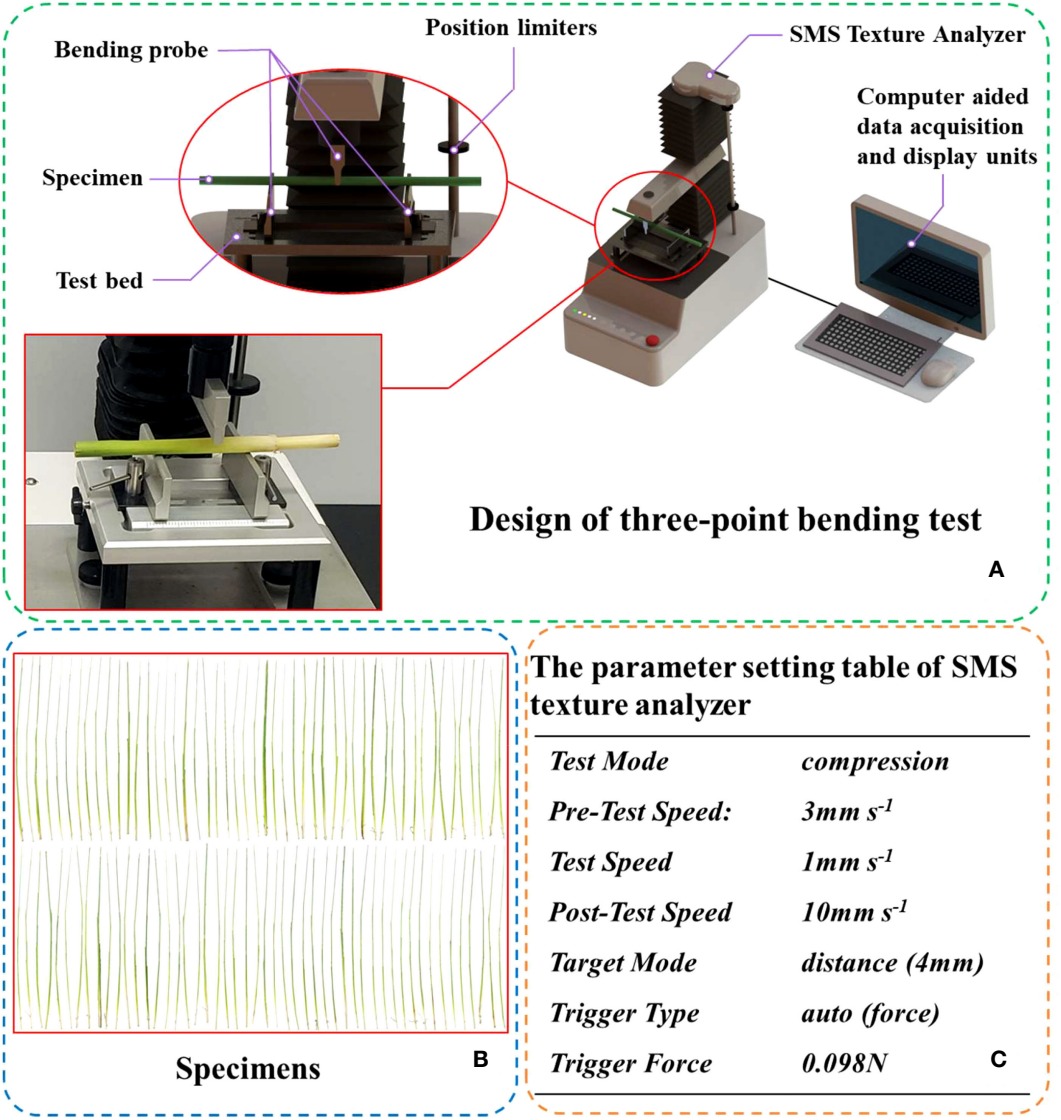
The set-up for the three-point bending test. **(A)** Design of three-point bending test, **(B)** All specimens used in the measurement of rice elastic modulus, **(C)** The parameter setting table of SMS texture analyzer.

**Figure 4:**
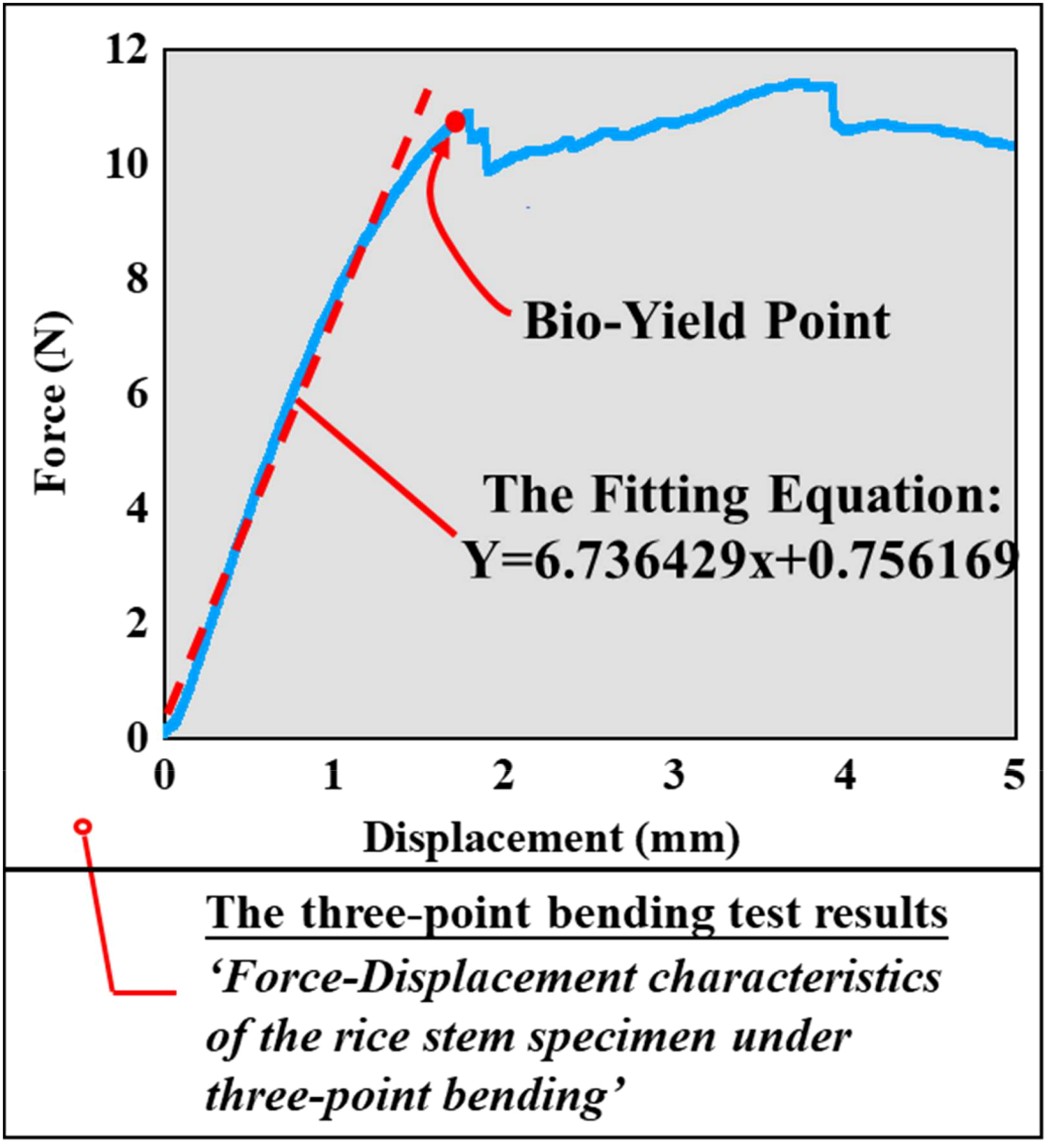
The force-displacement characteristics of the single rice stem specimen.

The three-point bending test method (**Figure 3A**) was adopted for the measurement of the elastic modulus of rice stems (Kokubo et al., 1989; Crook and Ennos, 1994). This method is based on the bending theory of the beam to measure the elastic modulus and indirectly calculates the elastic modulus by measuring the force-displace (Timoshenko and Gere, 1973). An SMS Texture Analyzer (TA.XT PlusC., Godalming, UK) was used for measurement. Remove blades from petioles and nodes before treatment. The elastic modulus of rice stem were measured and averaged using 100 selected rice plants (**Figure 4B**). The experimental design of the three-point bending method to measure the elastic modulus of rice is shown in **Figure 4A**. The setting parameters of SMS texture analyzer are shown in the **Figure 4C**.

The deformation characteristics of the rice stem specimen under quasistatic compressive stress were revealed by three-point bending tests (**Figure 3**). Under this compressive loading, the stem specimen displayed almost linear deformation behavior up to an initial bio-yield point. Beyond this point, permanent (plastic) deformation was observed and then the stem specimen collapsed. It is estimated that the force of the canopy-opening device on the rice stem cannot reach the bio-yield point of the rice material. So, the deformation of the rice in this process was simplified to elastic deformation. Therefore, it was only necessary to calculate the elastic modulus of the rice stem in this process, which represents the elastic modulus of the rice stem.

The calculation formula for elastic modulus E is shown in Formula (1):


(1) $$E=\frac{PL^{3}}{48YI}$$

where *E* is the modulus of elasticity, measured in N m^-2^; P is the magnitude of the bending force, measured in N; L represents the length of the test sample, measured in mm; Y represents the size of the bending deflection, measured in mm; and I is expressed as the magnitude of the moment of inertia of the section, measured in mm^4^.

The formula for calculating the moment of inertia of the section is shown in Formula (2).


(2) $$I=\frac{\pi }{4}[ab^{3}-(a-t)\cdot {\left(b-t\right)}^{3}]$$

In formula (2), a represents the long half-axis of the cross-section in millimeters (mm), while b represents the short half-axis of the cross-section, in mm. t represents the average wall thickness of the rice stem in mm.

The above formula (1) can be transformed into:


(3) $$E=\frac{1}{48}\cdot \frac{P}{Y}\cdot \frac{L^{3}}{I}$$

**Figure 3** displays the force-displacement curve of a rice stem collected using an SMS texture analyzer. Blue line before biological yield point represents the elastic deformation section of the rice stem. A straight-line fitting was performed on this section, as illustrated by the white line in the figure. The fitting equation, Y=6.736429x+0.756169, yielded a slope of k=6.736429, which corresponds to $\frac{P}{Y}$ in formula (3). The calculation parameters a, b, t, and L were obtained through experiments. By combining Formula (2) and Formula (3), the elastic modulus of each rice stem was calculated.

**Figure 5:**
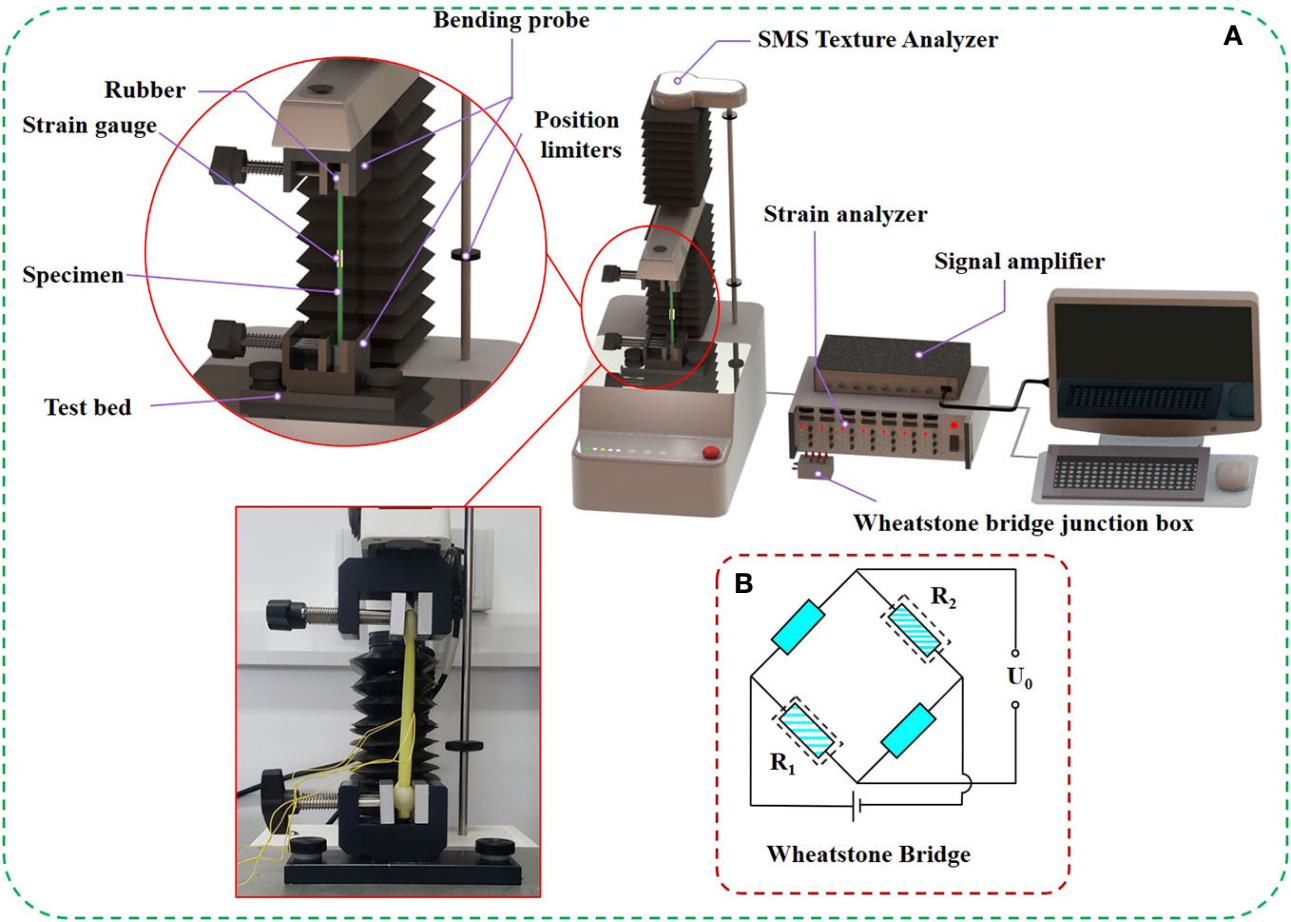
Measurement of poisson’s ratio in rice. **(A)** The set-up for the tensile and electrometric method testing, **(B)** The principle of electrical measurement.

Tensile tests and electrical measurements were used to conduct Poisson’s ratio test studies on rice stems. The axial strain of the surface of the test piece was measured by the tensile test, and the transverse strain of the surface of the test piece was simultaneously measured by the electrical measurement, to measure the Poisson’s ratio of the rice stem specimen (**Figure 5A**). Tensile tests were performed on an SMS texture analyzer. When preparing the rice stem specimen, the specimen should be straight, the two ends should be smoothed with sandpaper, and the two ends must be kept flush. The Poisson’s ratio of rice stem were measured and averaged using 100 selected rice plants. The size of the prepared specimen should be measured separately to reduce the error. Both ends of the specimen were wrapped with soft elastic rubber to reduce the damage of the fixture to the specimen (**Figure 5A**). Tension was applied to the specimen at a loading rate of 2 mm s^-1^. Tension and displacement changes were measured by a texture analyzer, and force-displacement-time records were obtained before specimen failure.

Electrical measurement is one of the methods commonly used in stress analysis experiments in engineering. The resistance strain gauge was attached to the measured point in the middle of the rice stem sample using a strong cyanoacrylate adhesive. The strain value generated while the rice stem specimen was subjected to the tensile test was converted into the resistance change of the strain gauge, and then the resistance variable of the strain gauge was measured by the strain analyzer, and the transverse strain value of the rice stem was directly converted and output (**Figure 5B**). The parameters of the strain gage used in this test are shown in **Supplementary Table 2**.

The calculation formula of Poisson’s ratio is shown in formula (4).


(4) $$\varepsilon =\left|\frac{\varepsilon_{p}^{\;\prime }}{\varepsilon_{p}}\right|$$

In Equation (4), ε is Poisson’s ratio; ε_p_ is the axial strain; ε_p_’ is the transverse strain.

### 2.2 Simulation condition and parameter setting

**Figure 6:**
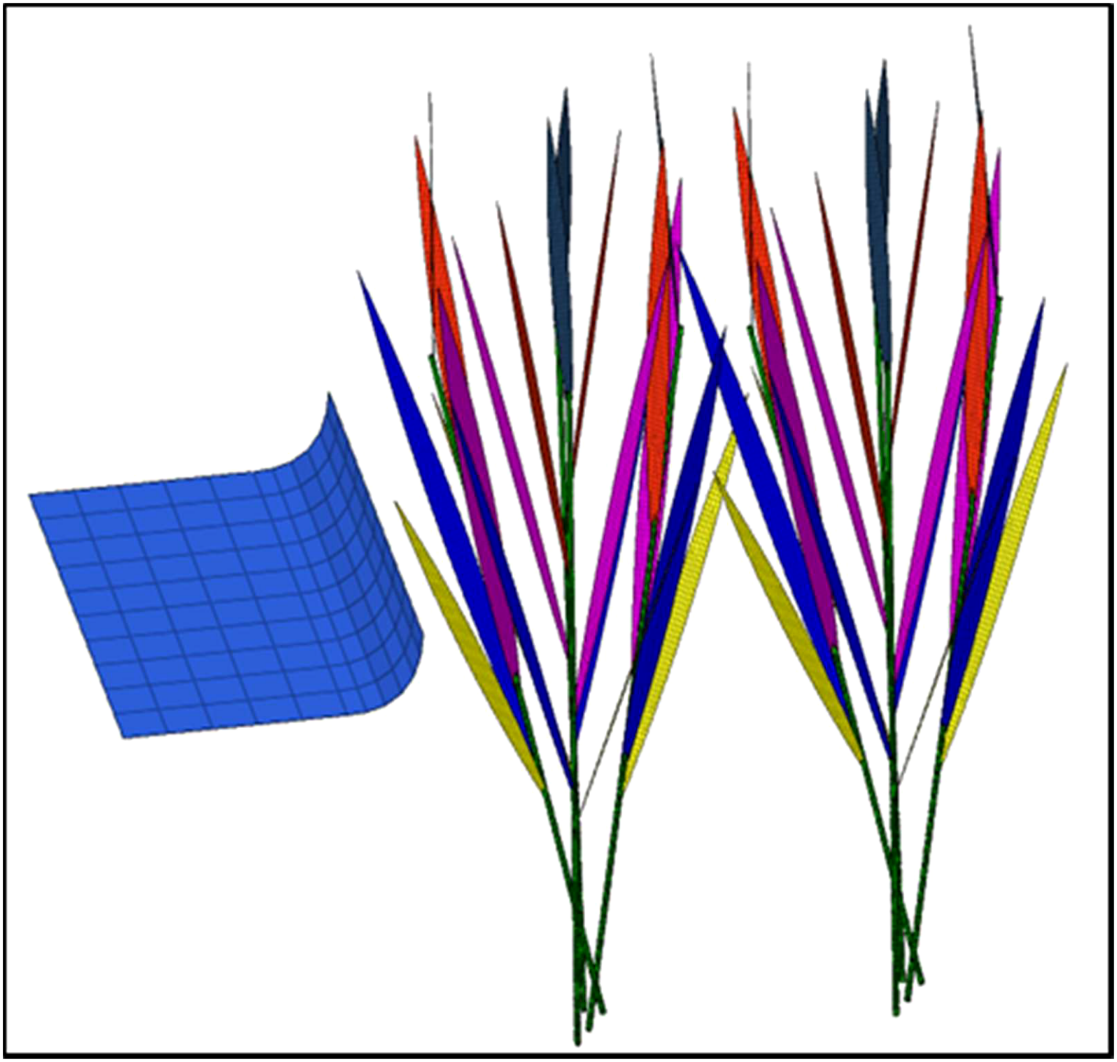
The canopy-opening 3D model and mesh generation.

In this study, a 3D model of rice canopy-opening dynamics was established by using SolidWorks software (SolidWorks 2019, Dassault Systems SolidWorks Corporation, USA). Each rice plant is assigned a random rotation angle to obtain the spatial position of the rice leaves in the population (**Figure 6**). The mesh of the model was divided by HyperMesh software (HyperMesh 10, Altair Engineering, Inc., Troy, MI). Using HyperMesh software, the material characteristics of the rice and canopy-opening devices in **Table 1** were established in their respective geometric models. Simultaneously, HyperMesh was utilized to pre-process the model of the rice canopy-opening process, including limitations, contact, speed, and so on. The process of rice canopy-opening was numerically simulated by LS-DYNA software (LS-DYNA 11.0, Livermore Software Technology Corporation, Livermore, CA, United States). LS-DYNA software was used as a dynamic analysis and was considered for the canopy-opening process simulation due to its capability of analyzing complex contacts and large dynamic deformations.

**Table 1:** Material properties of the rice and the canopy-opening device used in the simulation.

Material properties used in FEM-based simulation			
Materials	Modulus of elasticity (MPa)	Poisson’s ratio	Density (kg m^-3^)
Crop			
Rice (Longliangyou 2010)	158.8218	0.32	1815.69
The canopy-opening device			
Stainless steel	193000	0.310	7750

According to the basic principles of the finite element method, a 3D geometric model was meshed to develop a finite element model (**Figure 6**). The model includes eight rice plants (two clusters) and a canopy-opening device. To obtain more accurate numerical simulation results, shell mesh unit was used to preliminary divide the mesh (**Figure 6**). The rice leaves and the connection of leaves and stems were meshed sub-dividing, which are shown in **Figures 6**. After the sub-division was completed, the total finite element model had 31518 units and 33780 nodes. In HyperMesh, set the simulation time to 3 seconds, and make the rice stem’s bottom a fixed restriction. Contact is the sort of contact interaction attribute specified for two surfaces in HyperMesh, and hard contact is the usual behavior for this interaction.

To analyze the movement mechanism of the rice canopy, combinations of various canopy-opening heights and driving velocities were simulated through explicit dynamics simulation. Specifically, nine simulation combinations were created, consisting of three different heights of the canopy-opening device from the ground (0.5, 0.6, 0.7 m) and three different driving velocities (0.8, 1.2, 1.6 m/s). Explicit time integration typically requires smaller time steps than implicit time integration. Therefore, the LS-DYNA software’s explicit dynamics module was used to simulate these combinations. Result analysis was performed using LS-PrePost 4.5 (Livermore Software Technology Corporation, USA).

To conduct a numerical simulation of the rice canopy opening process, the HyperMesh software was used for pre-processing in this study. The rice plants were modeled as non-rigid bodies, while the canopy-opening device was modeled as a rigid body. To simulate the contact process, the automatic general contact type was selected. Subsequently, the LS-DYNA software was used to perform the numerical simulation of the rice canopy opening process. An explicit time integration method was used in the simulation process, with a smaller time step selected to obtain more accurate results. For the boundary conditions, the bottom of the rice stem was fixed with respect to rotation and transition in all directions, while the canopy-opening device was only allowed to translate along the X positive half-axis direction.

In the HyperMesh software, the MATL3 element type was used to model the rice plants. The rice plants were modeled as linear viscoelastic tissue. The canopy-opening device, which was folded from a 1000×500×2.6 mm steel plate, was modeled using the MATL20 element type with an elastic modulus of 193000 MPa and a density of 7750 kg m^-3^.To account for the non-rigid characteristics of rice plants, shell elements were used to model the rice plant in HyperMesh, with a mesh size of 0.001mm for element size and a mixed mesh type of triangular and quadrilateral elements. The final rice plant model consisted of 33648 nodes and 31408 elements. Similarly, shell elements were used to model the canopy-opening device, with an element size of 0.1 and 0.2 mm and quadrilateral elements used to generate a mesh consisting of 132 nodes and 110 elements.

### 2.3 Assessment verification

The high-speed camera (i-speed716, ix-cameras Company, Woburn, MA, USA) at maximum frame rate of 500,000 fps was utilized to track the movement trajectory of the rice stem under the action of the canopy-opening device in the real scene to evaluate the validity of the simulation results. Similarly, to the simulation combination, 9 actual combination situations were seted, and three distinct driving velocities (0.8, 1.2, 1.6 m s^-1^) of the canopy-opening device and different heights of the canopy-opening device from the ground (0.5, 0.6, 0.7 m) were examined. Each combination was repeated three times. Using ProAnalyst software (version 1.5.6.8, Xcitex Company, Woburn, MA, USA) to extract the three-dimensional coordinate motion trajectory of the rice canopy, and compared with the simulation results.

## 3 Result and discussion

### 3.1 Analysis of simulation results

**Figure 7:**
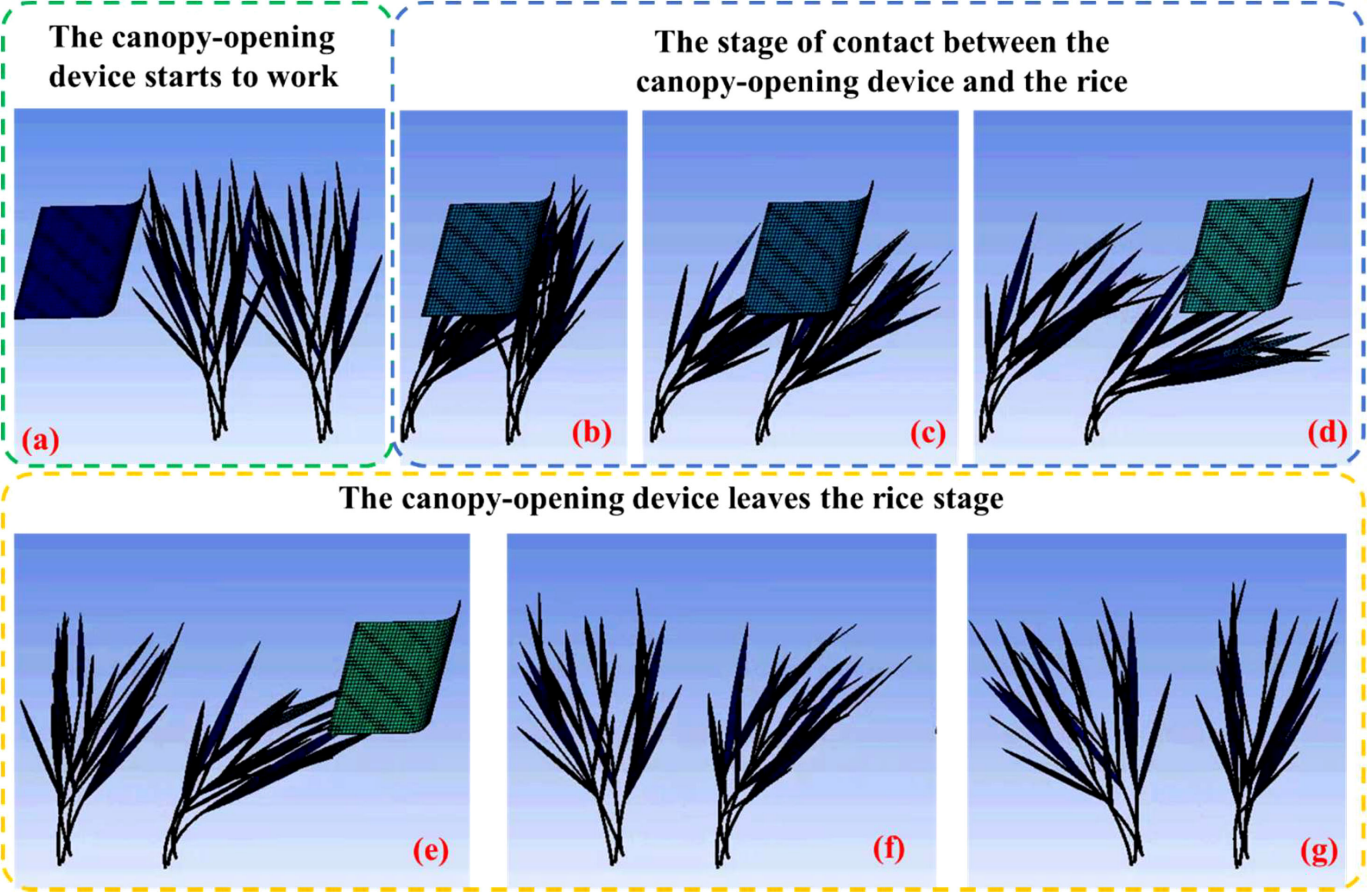
Simulation of a rice canopy-opening process. **(A)** The canopy-opening device starts to work, **(B-D)** The stage of contact between the canopy-opening device and the rice, **(E-G)** The canopy-opening device leaves the rice stage.

The model for the rice canopy-opening process was created in Hypermesh software and simulated using LS-DYNA software. A video recording of the rice canopy-opening process was also made. As an example of the simulation, the seven frames representing the canopy-opening process were taken from the video in chronological order and arranged according to the timeline, and divide the canopy-opening process into three stages, as shown **Figure 7**. In the first stage, the canopy-opening device has not been in contact with the rice, and the rice is in a static state. At this time, the upper canopy of the rice is relatively dense, and there is no obvious gap between the two clusters of rice (**Figure 7A**). In the second stage, the canopy-opening device moves to the right at a certain speed and begins to come into contact the rice, and the rice stems bend toward the forward direction of the canopy-opening device under the action of the canopy-opening device (**Figure 7B**). When the canopy-opening device entirely leaves the first cluster of rice, it begins to rebound in the opposite direction of the canopy-opening device’s forward motion (**Figure 7C**). Because of the varied positions, the rice stems that first encountered the canopy-opening device rebounded earlier than those that came later. A gap emerged in the canopy of the first cluster of rice at this time (**Figure 7D**). In the third stage, the canopy-opening device completely leaves the rice (**Figure 7E**). The first cluster of rice has rebounded in the opposite direction to the forward direction of the canopy-opening device (**Figure 7F**), the second cluster of rice is about to start to rebound, and there is a large gap between the two clusters of rice (**Figure 7G**).

In the explicit dynamics simulation of the canopy-opening process, not only the contact process between the canopy-opening device and the rice stem, but also the rebound oscillation motion process of the rice stem after the canopy-opening device leaves were also considered.

**Figure 8:**
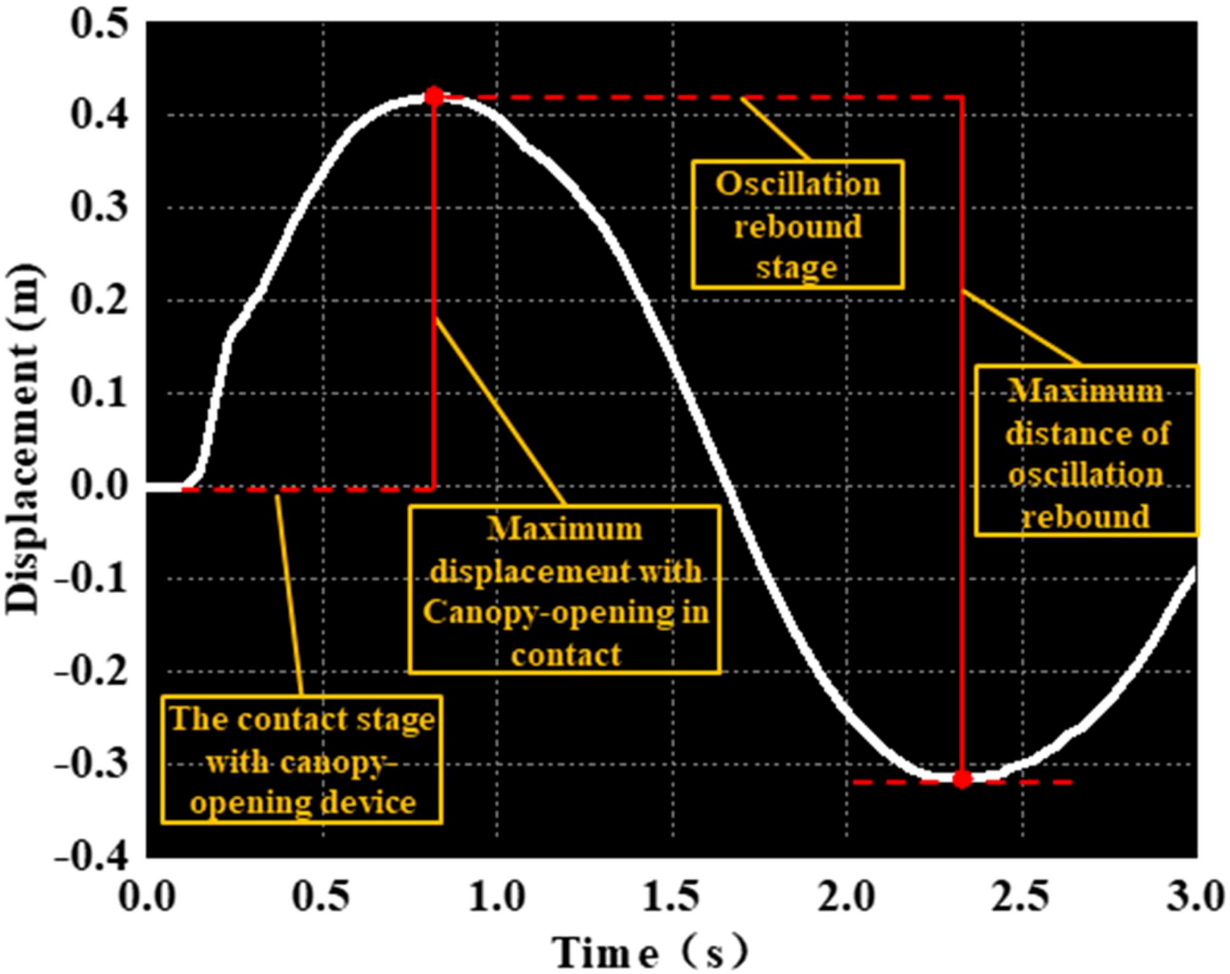
Schematic diagram of the characteristics of the canopy-opening process.

To analyze the simulation results of the rice canopy opening process, the process was divided into two stages: the contact stage and the oscillation stage. The stage in which the canopy-opening device makes contact with the rice stem was defined as the contact stage, while the stage in which the rice stem rebounds and oscillates after the device leaves was defined as the oscillation stage. It can be seen from **Figure 8** that when the canopy-opening device starts to contact with the rice stem, the rice stem is displaced by force, and reaches the maximum displacement in the contact stage when it leaves the canopy-opening device. As the canopy-opening device moved away from the rice stem, the rice stem began to rebound and entered the oscillation stage, and the displacement and the amplitude of the oscillation gradually decreased.

**Figure 9:**
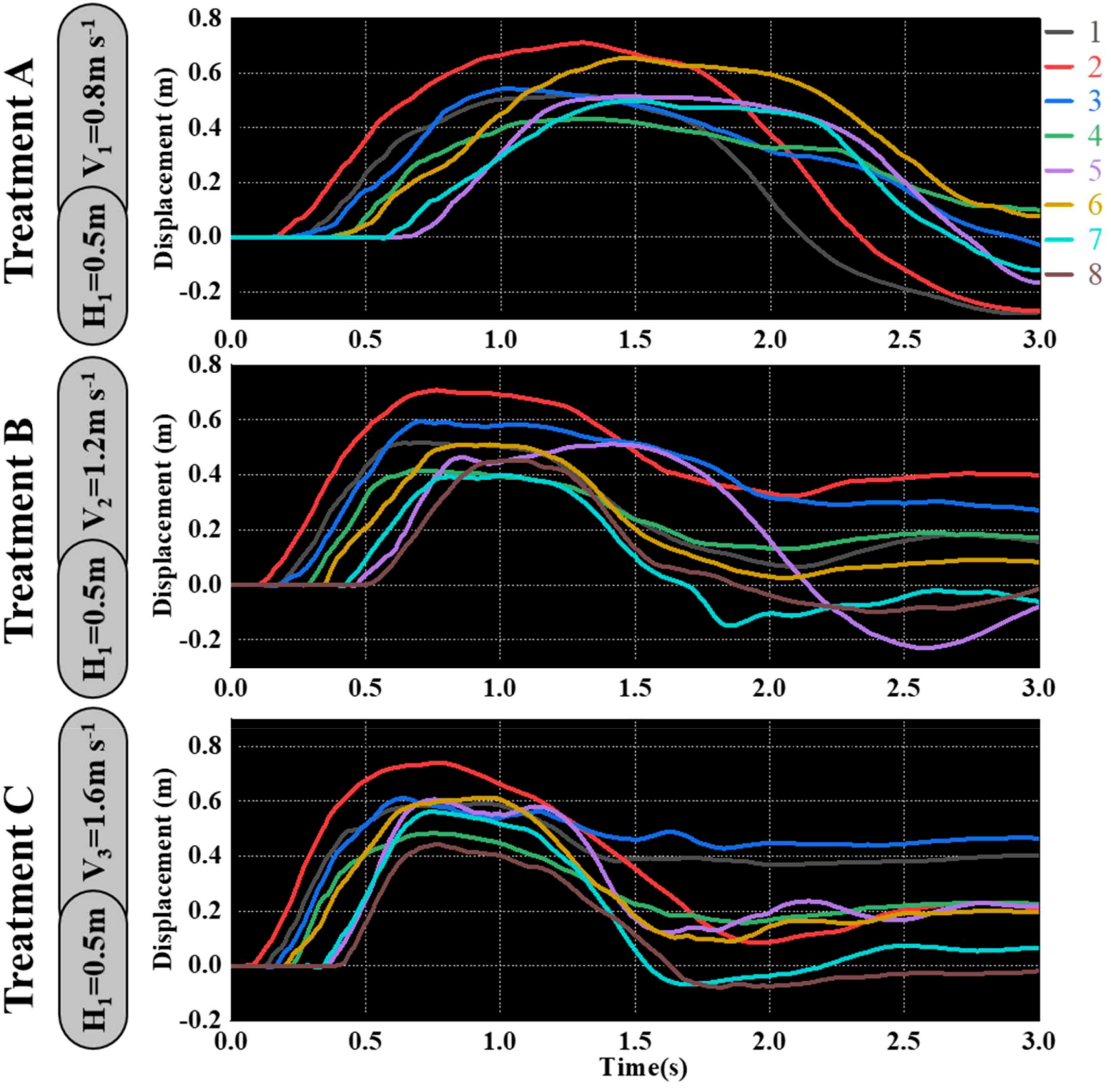
When the canopy-opening device is 0.5m above the ground, the relationship between time and displacement of rice stems without the effect of velocity. (1) 1~8 are rice plants in different positions.

**Figure 10:**
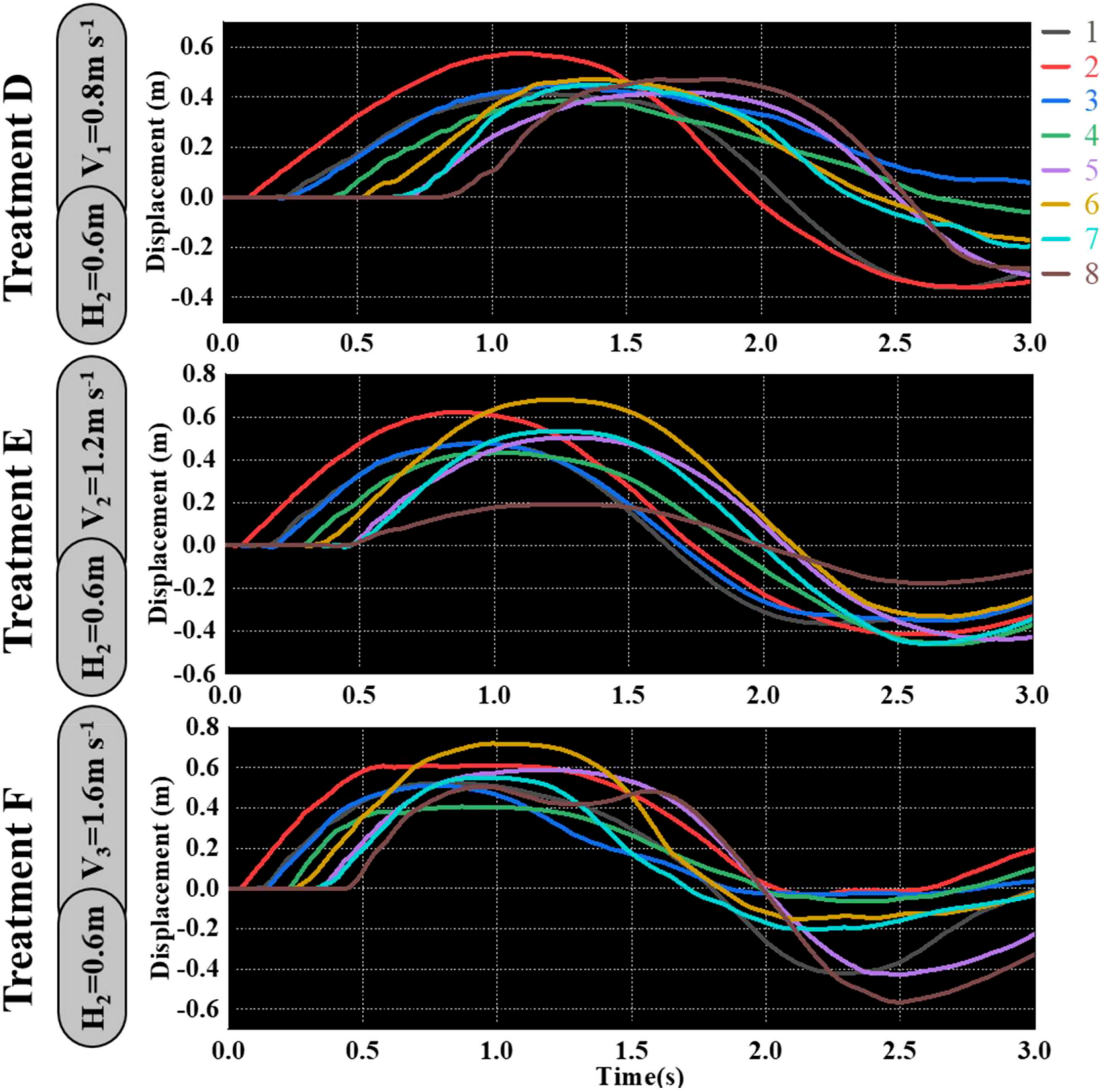
When the canopy-opening device is 0.6m above the ground, the relationship between time and displacement of rice stems without the effect of velocity.

**Figure 11:**
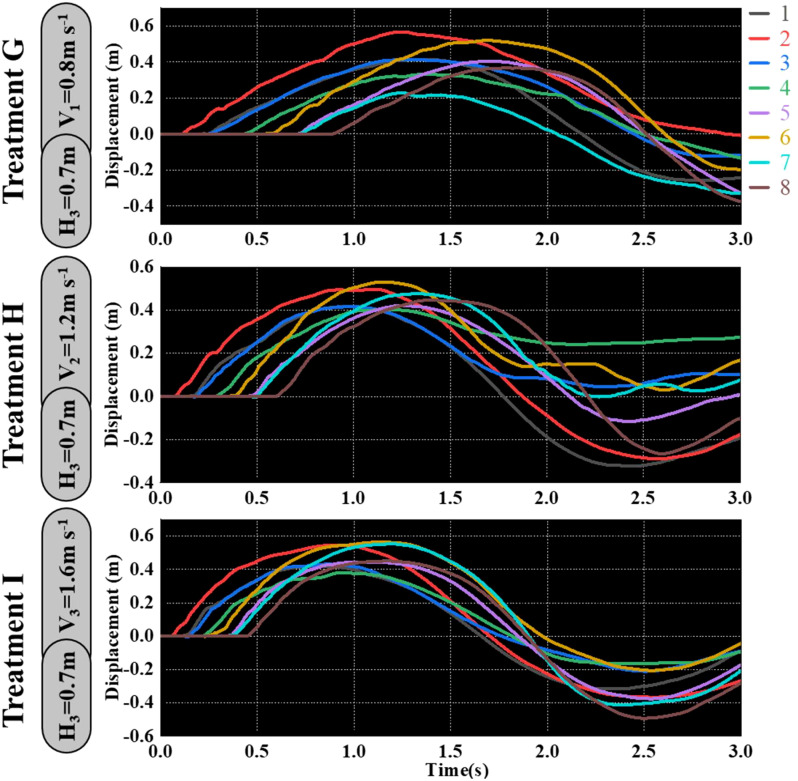
When the canopy-opening device is 0.7m above the ground, the relationship between time and displacement of rice stems without the effect of velocity.

As shown in **Figures 9**–**11**, the fluctuation of rice stem displacement with time while keeping the same height of the canopy-opening device and increasing the velocity of the canopy-opening device. When the height of the canopy-opening device is 0.5m and its velocity is 0.8m/s, the rice stems experience its maximum displacement roughly one second after coming into contact with the canopy-opening device (**Figure 9**). The faster the velocity of the canopy-opening device, the sooner the rice stems will reach the maximum displacement in the contact stage. This conclusion is also applicable when the canopy-opening device height is 0.6 m and 0.7 m (**Figures 10**, **11**). Furthermore, when the driving velocity of the canopy-opening device increases, the oscillation amplitude of the rice stems increases and the oscillation period decreases.

**Figure 12:**
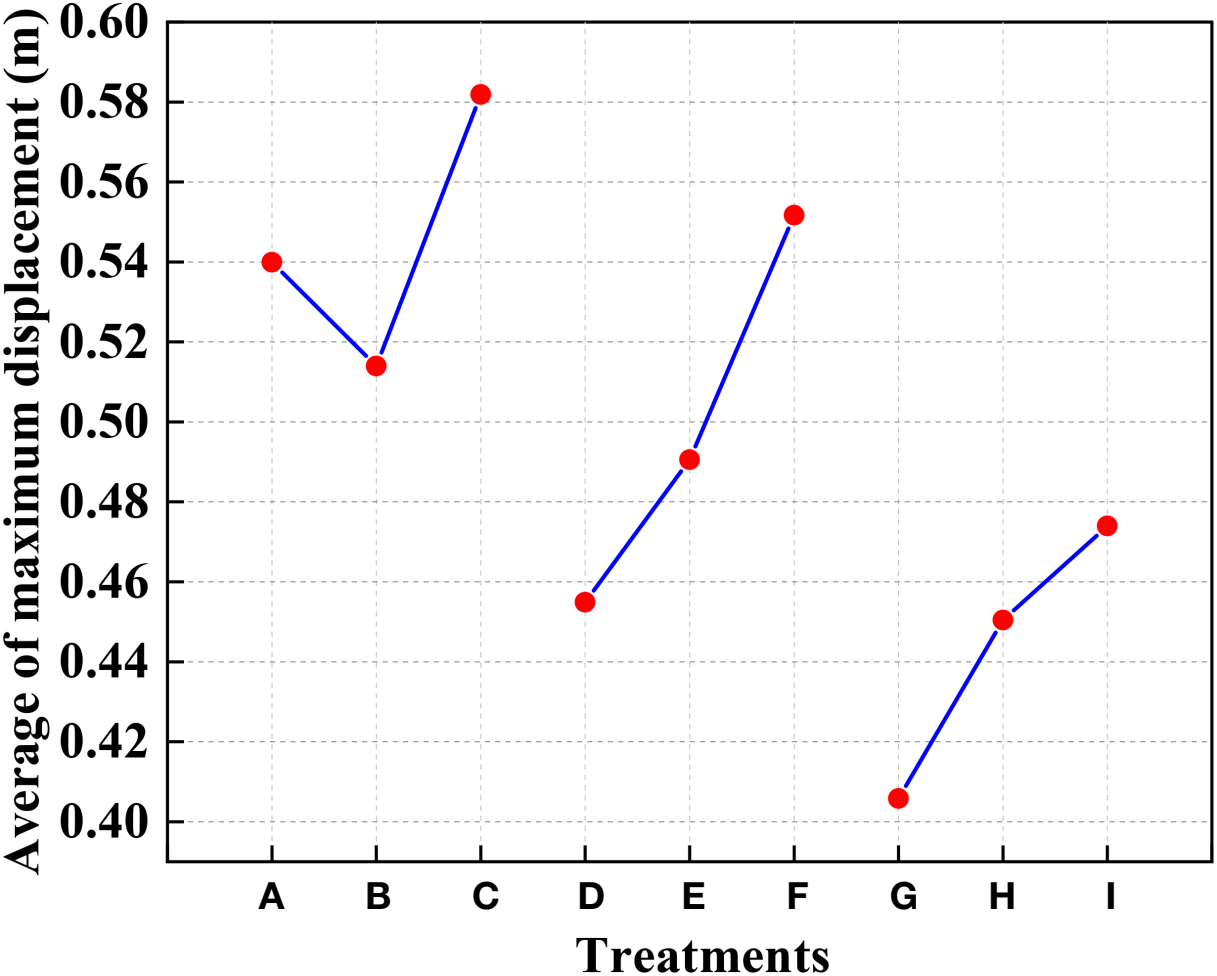
The average of the maximum displacement of rice stems under different treatments in the contact stage. (1) **(A-I)** are different experimental treatments in **Figures 9**–**11**.

**Figure 12** shows that the highest displacement of the contact stage between the rice stems and the canopy-opening device may be created when the deriving velocity of the canopy-opening device was 1.6 m s^-1^. From **Figure 12**, it can be concluded that, when the deriving velocity of the canopy-opening device is constant, the maximum displacement of the rice stem during the contact stage decreases as the height of the canopy-opening device above the ground increases. This is because the lower the height of the canopy-opening device above the ground, the greater the bending of the rice stem, and the larger the displacement. If the device continues to lower and the height above the ground decreases further, the displacement may increase, but the canopy-opening device may also damage the rice stem. When the canopy-opening device was 0.7 m above the ground, it is positioned in the upper portion of the rice canopy, and the contact and action time between the canopy-opening device and the rice stem was short, a considerable displacement could not be obtained.

When the height of the canopy-opening device above the ground is constant, the overall trend of the maximum displacement of the rice stem during the contact stage increases as the deriving velocity increases. It is obvious that reducing the deriving velocity will decrease the impact energy of the canopy-opening device on the rice stem. However, when the canopy-opening device is 0.5m above the ground, the rice stem shows a trend of first decreasing and then increasing in the oscillation stage as the deriving velocity increases.

**Figure 13:**
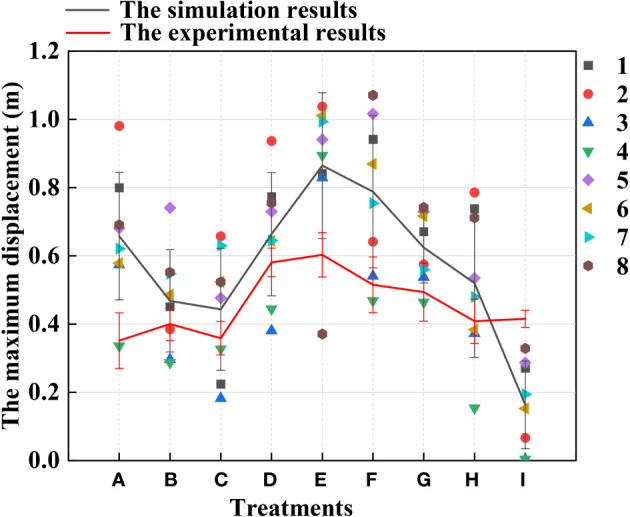
Comparison of the difference between the experimental results and the simulation results in the oscillation stage. (1) The point in the Figure is the maximum displacement of the rice stems in the oscillation stage under the simulation result.

**Figure 13** shows the maximum displacement of rice stem during the oscillation stage under different treatments and the maximum displacement of rice stem measured by high-speed photography experiments. It can be seen that when the canopy-opening device is 0.6m above the ground and the deriving velocity of the canopy-opening device is 1.2m s^-1^ (Treatment E), the maximum displacement of rice stem is the largest, approximately 1.04m.

Under the same deriving velocity of the canopy-opening device, the maximum displacement of rice stem generated by oscillation stage first increases and then decreases with the increase of the height of the canopy-opening device. When the height is 0.6m, the maximum displacement is greater than that of other heights. At the same height, increasing the deriving velocity of the canopy-opening device cannot obtain a better maximum displacement of rice stem. On the contrary, when the height of the canopy-opening device is 0.5m and 0.7m, the maximum displacement of rice stem generated by the canopy-opening device decreases with the increase of deriving velocity. When the height of the canopy-opening device is 0.6m, the maximum displacement of rice stem generated by the canopy-opening device increases first and then decreases with the increase of deriving velocity, reaching the maximum value at the deriving velocity of 1.2m s^-1^.

**Figure 14:**
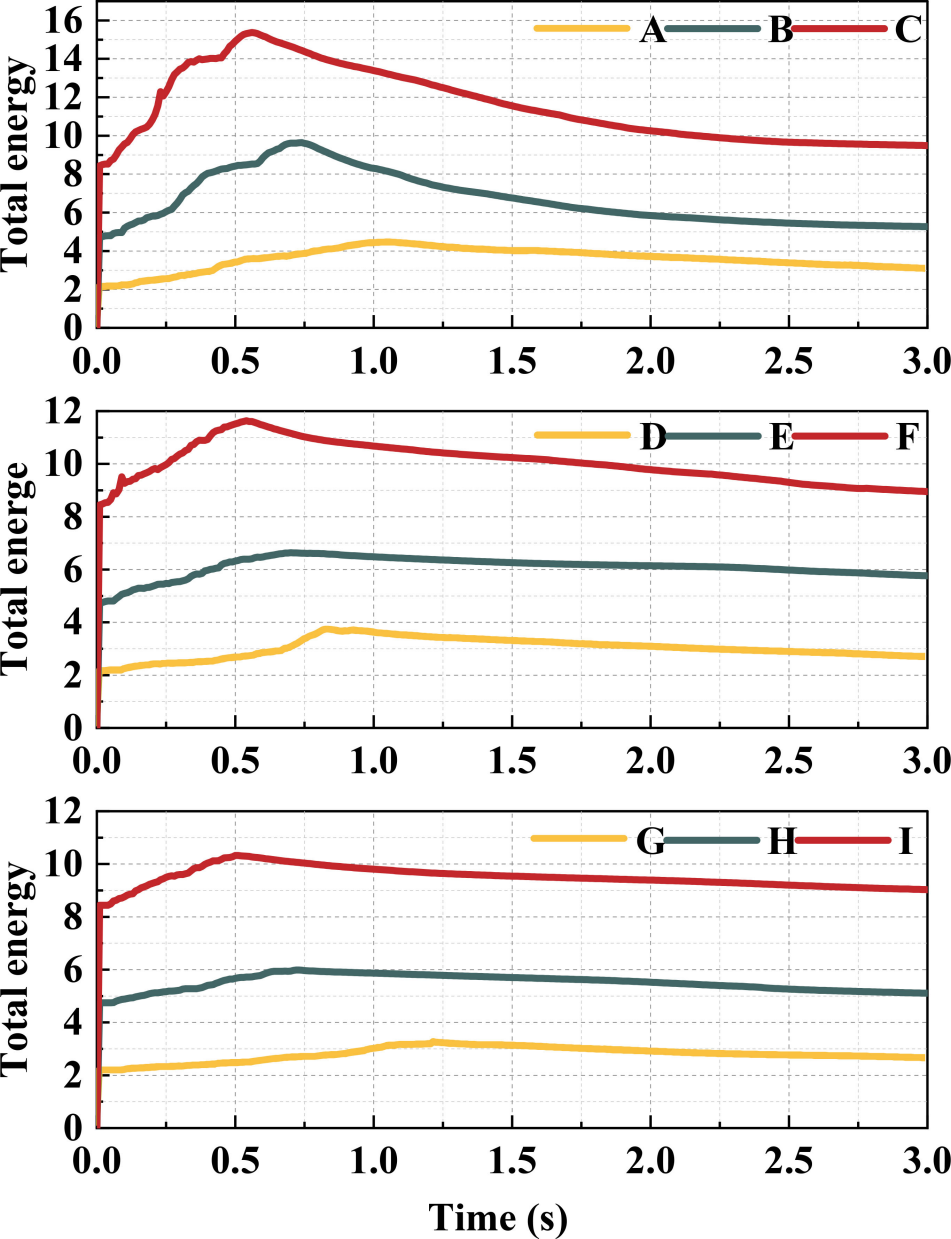
The total energy of canopy opening model under different treatments.

**Figure 14** provides the trend of the total energy of rice canopy model under different treatments. It can be seen from the **Figure 14** that the total energy of the model increases first and then decreases with the increase of time; under the same height of the canopy-opening device, the larger the deriving velocity, the higher the total energy of the model; the larger the deriving velocity, the earlier the model reaches the maximum total energy; under the same deriving velocity, the higher the height, the lower the total energy of the model. However, due to the interaction between the canopy-opening device and rice stem during the oscillation stage, the total energy of the model decays rapidly after reaching the maximum value when the deriving velocity is high. It can be seen from **Figure 14** that under Treatment E, the total energy of the model decays slower after reaching the maximum value. Therefore, when the canopy-opening device is 0.6m above the ground and the deriving velocity is 1.2m s^-1^, the interference intensity on rice stem during the oscillation stage is the highest, and a larger canopy gap can be obtained.

### 3.2 Comparison of test results and simulations

**Figure 15:**
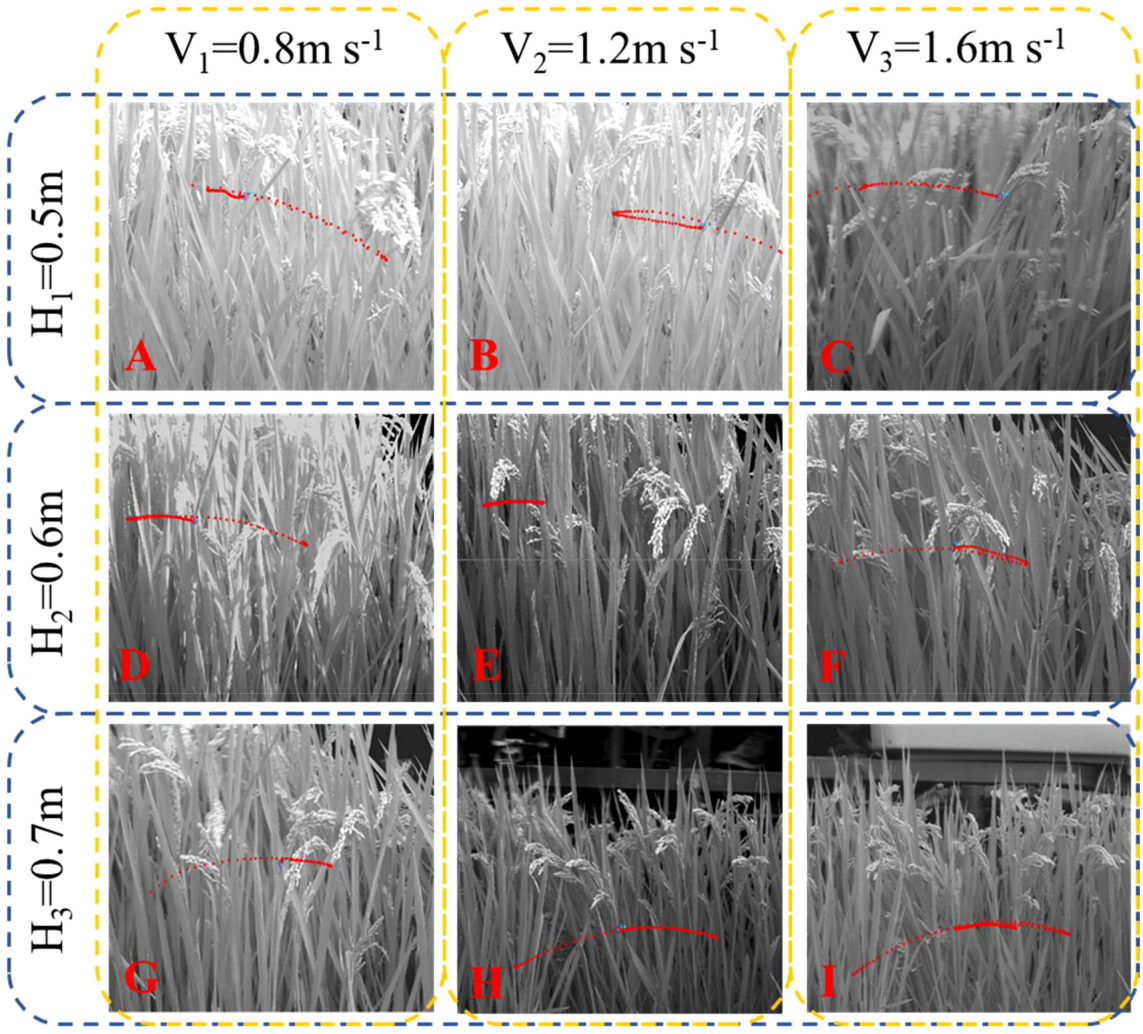
Maximum displacement of rice stems during oscillation stage captured by high-speed photography.

Simulation results were verified by high-speed testing. The movement of rice plants due to the action of the canopy-opening was recorded using high-speed photography (**Figure 15**). Calibrate the movement track of the rice stem by the length of the known black and white area in the calibration group. According to the track plots of rice plants, the average value of the maximum displacement of the rice stems after being acted by the canopy-opening device was calculated.

**Figure 16:**
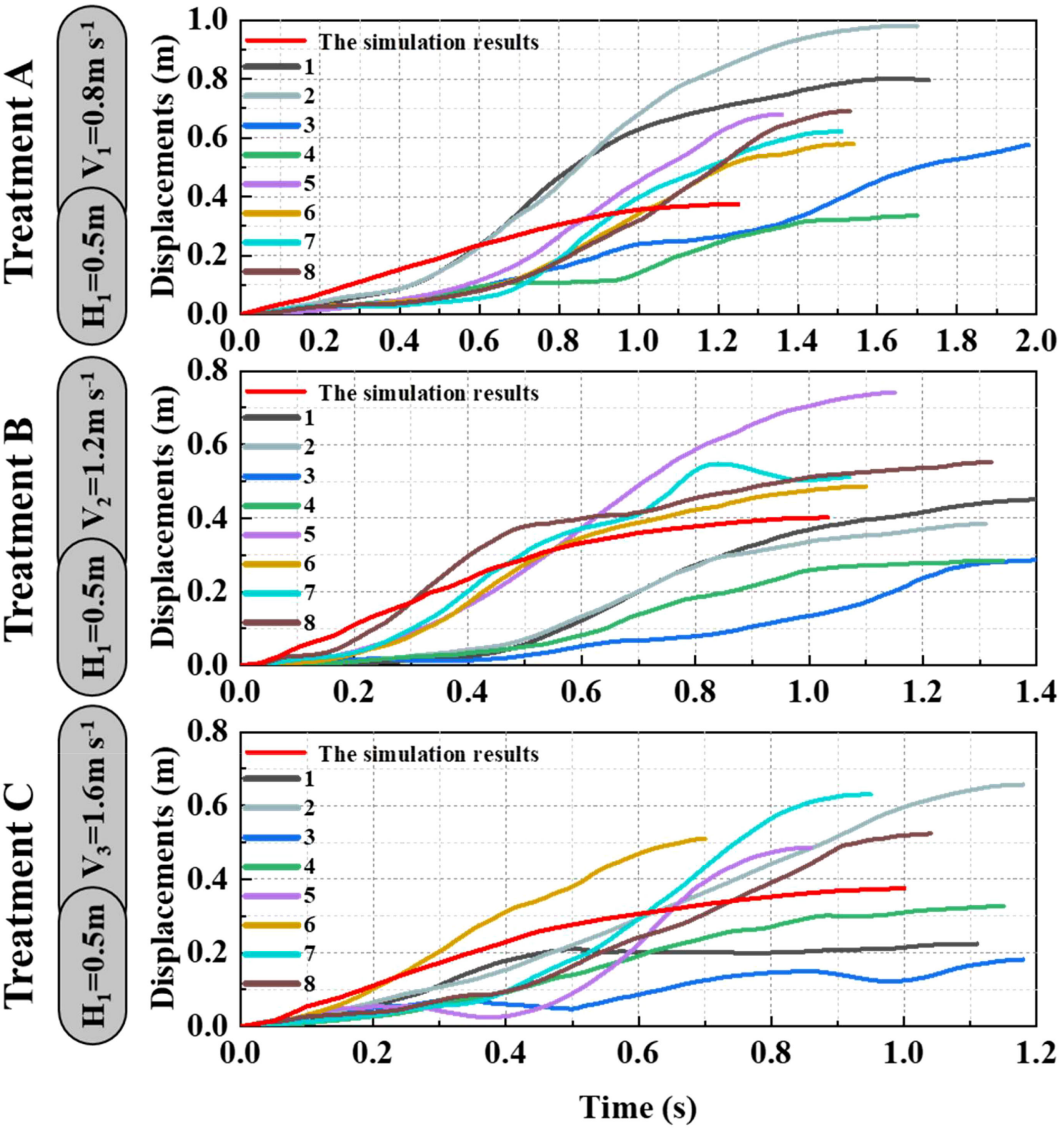
The simulation and experimental results during the oscillation stage when the height of the canopy-opening device from the ground was 0.5 m. (1) 1~8 are rice plants in different positions.

**Figure 17:**
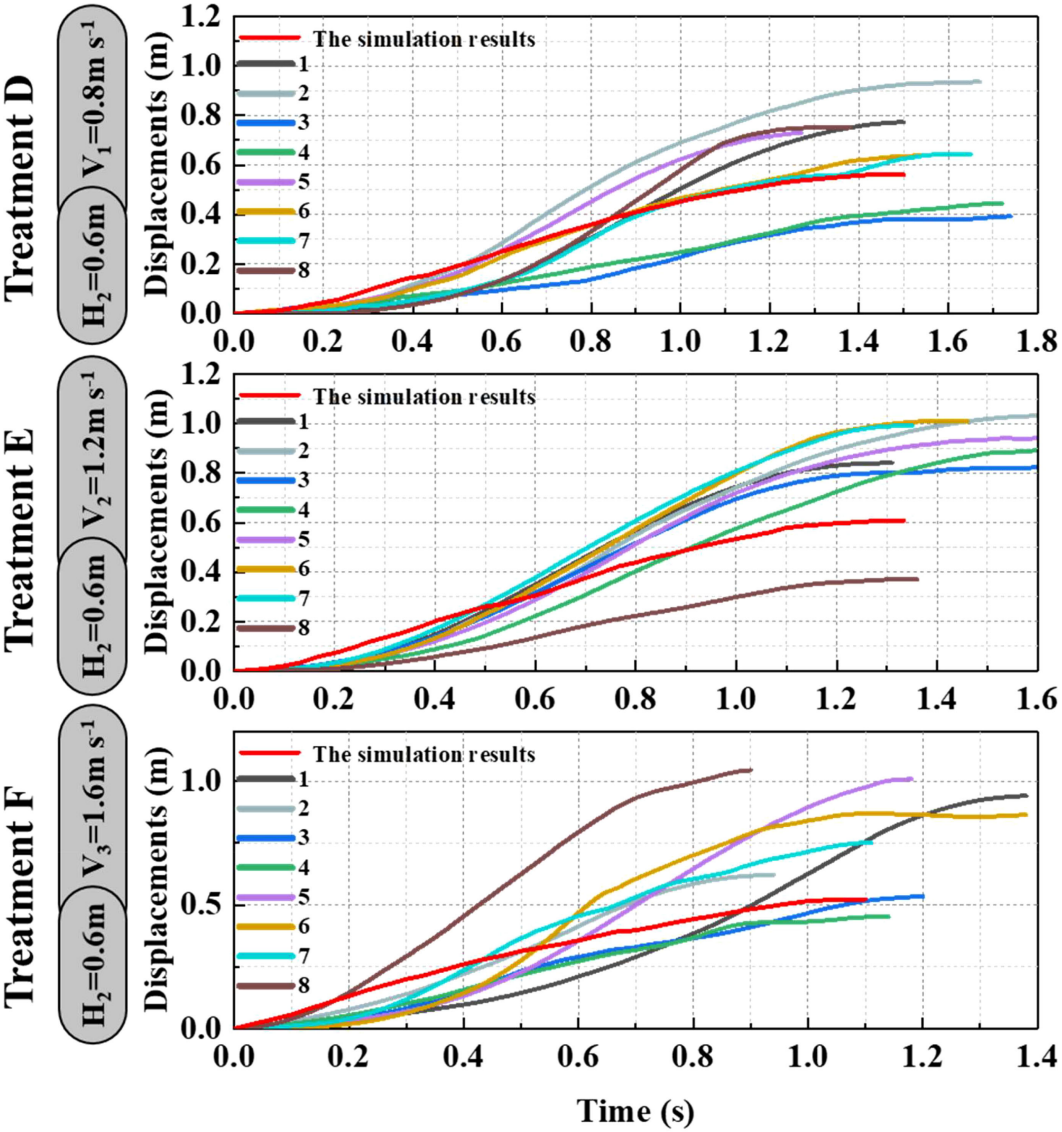
The simulation and experimental results during the oscillation stage when the height of the canopy-opening device from the ground was 0.6 m.

**Figure 18:**
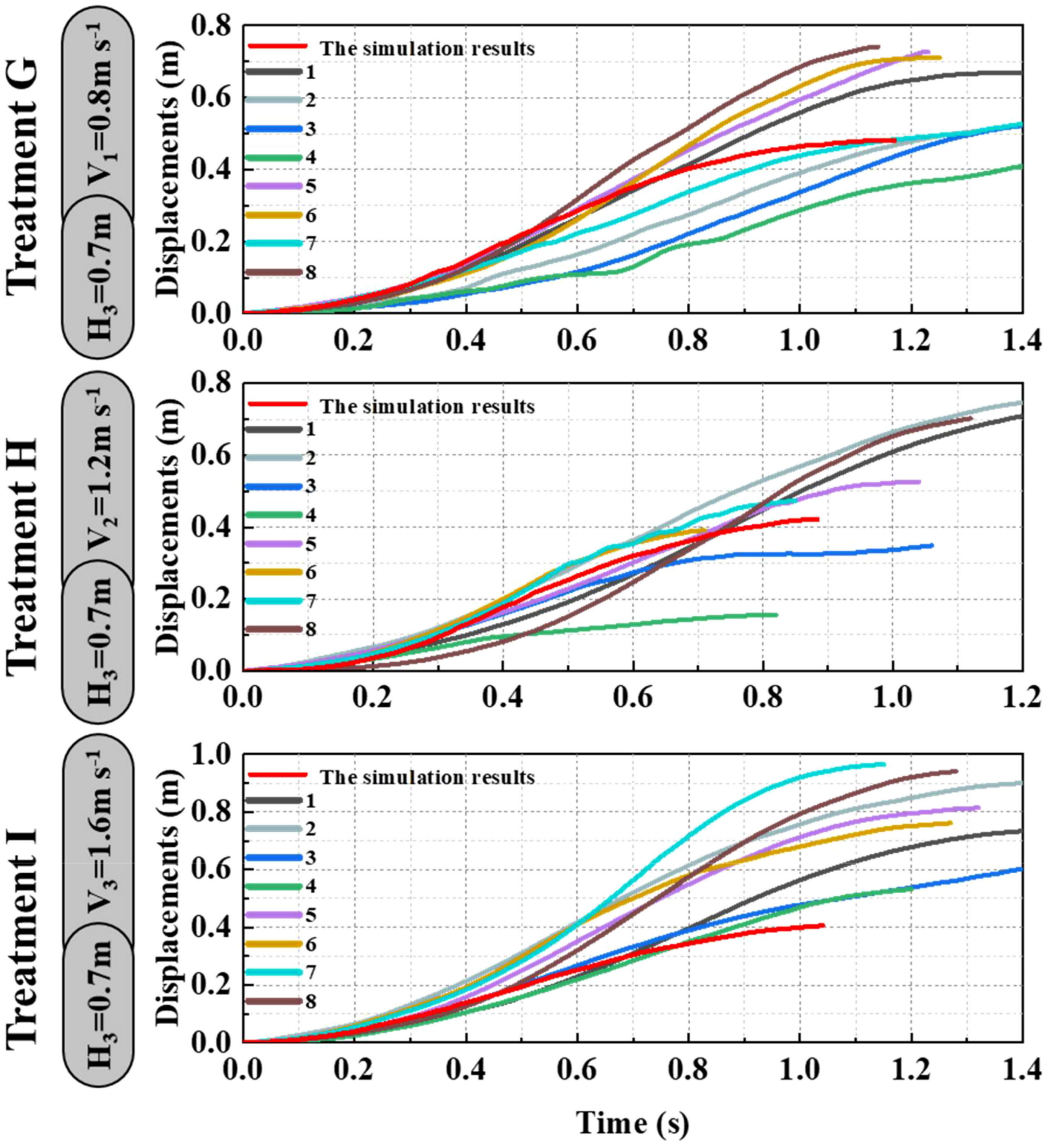
The simulation and experimental results during the oscillation stage when the height of the canopy-opening device from the ground was 0.7 m.

When the canopy-opening device leaves rice plants, rice plants exhibited a rebound-oscillation movement due to its elasticity. **Figure 13** shows the average value of the greatest displacement of rice stems in the simulation results and experimental results during the oscillation stage. **Figure 16** compares the simulated and measured values during the oscillation stage when the height of the canopy-opening device from the ground was 0.5 m. **Figures 17**, **18** show similar comparisons when the height of the canopy-opening device from the ground was 0.6 m and 0.7 m respectively. It showed that the relative difference between the simulation data and the test data was consistent. Because the field test was affected by too many uncontrollable factors (such as wind force, and wind direction), the occlusion of blades also affects the test data. Therefore, there are large fluctuations in the test data. However, values similar to the measured values can still be found in the simulation values. Comparison of the maximum displacement of rice stem in the oscillation stage between high-speed photography experiment results and simulation results (**Figure 13**) and the errors shown in **Table 2**, the minimum and maximum errors in predicting the maximum displacement in the oscillation stage are 12.12% and -162.50%, respectively. The error caused under Treatment I is the largest, which may be due to the unstable relative height between the canopy-opening device and the rice under field conditions, resulting in a larger maximum displacement of the rice stems in the oscillation stage. If we exclude the extreme condition of Treatment I (with the deriving velocity and high height of the canopy-opening device), the correlation coefficient between the simulation results and the high-speed photography experiment results is 0.733, indicating a good correlation between the simulation results and the high-speed photography experiment results.

**Table 2:** The results of rice canopy opening in the oscillation stage.

Treatments	Average of maximum displacement in oscillation stage (m)		
	Simulation	Experimental	Error (%)
**A**	0.66	0.35	46.97
**B**	0.47	0.40	14.89
**C**	0.44	0.36	18.18
**D**	0.66	0.58	12.12
**E**	0.86	0.60	30.23
**F**	0.79	0.52	34.18
**G**	0.62	0.49	20.97
**H**	0.52	0.41	21.15
**I**	0.16	0.42	-162.50

## 4 Conclusion

The main purpose of this study is to simulate the rice canopy opening process using an explicit dynamic method and investigate its disturbance mechanism. The morphological and material parameters of rice were measured, and the simulation model of the rice canopy opening process was established. The canopy opening process was simulated using an explicit dynamic method. When analyzing the simulation results, the canopy opening process was divided into two stages: contact stage and oscillation stage.

The simulation results show that in the contact stage, the maximum displacement of the rice stem increases with the decrease in the height of the canopy opening device from the ground and the increase in deriving velocity. In the oscillation stage, there is a critical value for the height and deriving velocity of the canopy-opening device, and heights and deriving velocities that are too high or too low cannot increase the maximum displacement of the rice stem. When the height of the canopy-opening device is 0.6 m and the deriving velocity is 1.2 m s^-1^, the disturbance intensity of the canopy-opening device to the rice stem is the maximum in the oscillation stage. The total energy of the model increases with the decrease in the height of the canopy-opening device and the increase in its deriving velocity, but the increase in deriving velocity also increases the rapid decay of the total energy of the model. High-speed photography experimental results show that there is a certain error between the simulation results in the oscillation stage and the high-speed photography experimental results. However, if extreme processing is removed, the simulation results and experimental results show a strong correlation, with a correlation coefficient of 0.733. However, the error between the simulated rice stem displacement and the observed displacement is large during the process of the canopy opening. This is because the canopy opening process is a very complex physical phenomenon influenced by many factors, such as the growth status of the rice and the material properties of the rice stem. Therefore, even with accurate physical parameters and fine grid division in the simulation, it is difficult to completely replicate the actual situation. However, increasing the number of rice plants in the simulation model can better reflect the interaction between rice plants in reality, which maybe help to reduce the error between the simulation and real results. This is also our next work.

## Data availability statement

The original contributions presented in the study are included in the article/**Supplementary Material**. Further inquiries can be directed to the corresponding author.

## Author contributions

The contribution of L-LJ is conceptualization, methodology, data curation, writing original draft and writing review and editing. The contribution of X-HW is methodology, writing review and editing, validation, and funding Support. The contribution of QS is data curation and methodology. The contributions of FW is methodology. All authors contributed to the article and approved the submitted version.
