# Supplementary material for: Study on the movement mechanism of rice stem under the action of canopy-opening device based on explicit dynamics simulation
Source: Front Plant Sci. 2023 Oct 24;14:1252247. doi: 10.3389/fpls.2023.1252247 (PMC10635423; doi:10.3389/fpls.2023.1252247)
Supplement: Supplementary file 1 [file DataSheet_1.pdf]

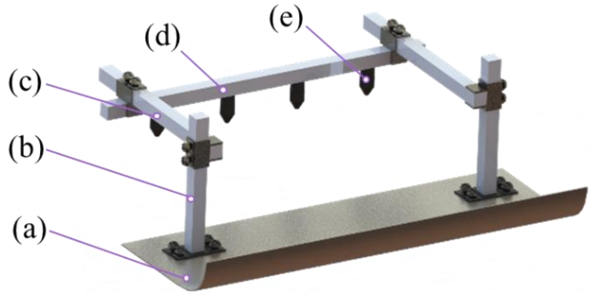

(a) The canopy-opening device, (b) Adjust the height between the canopy-opening device and the spray boom, (c) Adjust the horizontal distance between the canopy-opening device and the spray boom, (d) The spray boom, (e) The nozzle.

Figure.1. 3D model of the canopy-opening device.

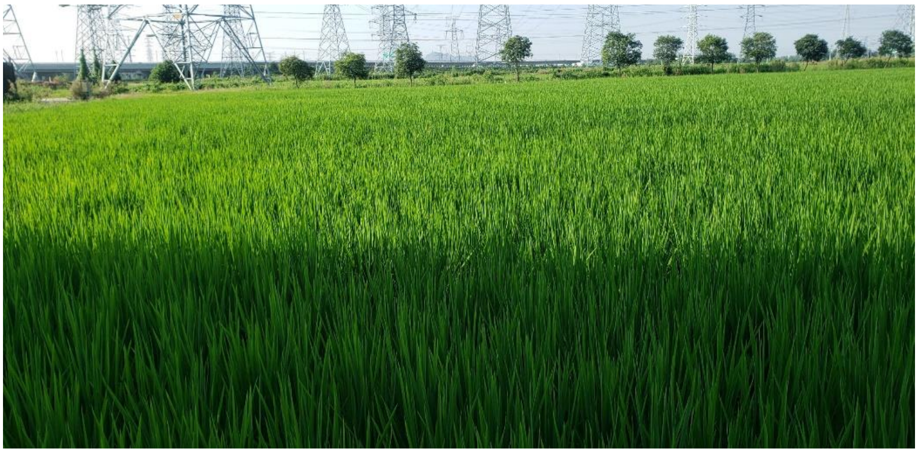

Figure.2. Rice sampling field.

Table.1. The morphological structure parameters of rice.

Average measurement of the whole rice					
**Dimensions**	**Average measurement**				
	**Leaf A**	**Leaf B**	**Leaf C**	**Leaf D**	**Leaf E**
**Distance from leaf root to bottom of stem (mm)**	**251.26**	**290.85**	**381.61**	**535.82**	**757.41**
**Leaf inclination (°)**	**20.58**	**19.02**	**15.69**	**11.16**	**8.86**
**Leaf length (mm)**	**455.38**	**559.22**	**535.00**	**459.72**	**371.15**
**Leaf area (cm^2)**	**91.75**	**120.43**	**123.89**	**110.29**	**92.62**
**Leaf width (cm)**	**2.84**	**3.08**	**3.38**	**3.44**	**2.22**
**Stem stalk diameter(mm)**	**9.40**	**9.27**	**8.19**	**6.62**	**4.56**
**Horizontal deflection angle from the first leaf(°)**	**128.57**	**44.00**	**186.63**	**129.47**	
**Plant height(m)**	**0.758**				
**Volume*(cm^3)**	**8.05**				
**Mass(g)**	**14.62**				

Whole rice volumes have been calculated to frustum of a cone specimen density(specimen density:1815.69 kg m^{-3})

Table.2. Parameters of strain gage.

Type	BE120-3AA-P500
Resistance (Ω)	120.1±0.1
Gage factor	2.22±1%
Operating temperature range (°C)	-30~+80
sensing grid element size Long × Width (mm)	2.8×2.0
Strain gage substrate size Long × Width (mm)	6.4×3.5
